# Cycling Isokinetic Peak Force Explains Maximal Aerobic Power and Physiological Thresholds but Not Cycling Economy in Trained Triathletes

**DOI:** 10.3390/jfmk9040273

**Published:** 2024-12-13

**Authors:** Felipe Giancáspero-Inostroza, Carlos Burgos-Jara, Carlos Sepúlveda, Danni Haichelis, Roberto Meneses-Valdés, Ignacio Orizola-Cáceres, Hugo Cerda-Kohler

**Affiliations:** 1 Unidad de Fisiología del Ejercicio, Centro de Innovación, Clínica MEDS, Santiago 7550615, Chile; felipe.giancaspero@meds.cl (F.G.-I.); carlos.burgos@meds.cl (C.B.-J.); carlos.sepulveda@meds.cl (C.S.); danni.haichelis@meds.cl (D.H.); ignacio.orizola@meds.cl (I.O.-C.); 2 Magister en Ciencias de la Salud y el Deporte, Escuela de Kinesiología, Facultad de Medicina, Universidad Finis Terrae, Santiago 7501015, Chile; 3 Unidad de Ciencias Aplicadas al Deporte, Instituto Nacional de Deportes, Santiago 7750332, Chile; 4 The August Krogh Section for Molecular Physiology, Department of Nutrition, Exercise and Sports, University of Copenhagen, Universitetsparken 13, 2100 Copenhagen, Denmark; roberto.meneses@ug.uchile.cl; 5 Departamento de Educación Física, Deporte y Recreación, Facultad de Artes y Educación Física, Universidad Metropolitana de Ciencias de la Educación, Santiago 7750332, Chile; 6 Laboratory of Psychophysiology and Performance in Sports and Combats, Postgraduate Program in Physical Education, School of Physical Education and Sport, Federal University of Rio de Janeiro, Rio de Janeiro 21941-853, Brazil

**Keywords:** crank cycling, aerobic performance, isokinetic strength

## Abstract

**Background**: Assessments of muscle strength help prescribe and monitor training loads in cyclists (e.g., triathletes). Some methods include repetition maximum, joint isokinetic tests, and indirect estimates. However, their specificity for cycling’s dynamic force application and competitive cadences is lacking. This study aims to determine the influence of the cycling isokinetic peak force (cIPF) at different cadences on aerobic performance-related variables in trained triathletes. **Methods**: Eleven trained male athletes (33 ± 9.8 years, 173.1 ± 5.0 cm height, 73.9 ± 6.8 kg body mass, and ≥5 years of triathlon experience) were recruited. Maximal oxygen consumption (VO_2_ max), ventilatory thresholds (i.e., VT1 and VT2), and cIPF were assessed. cIPF testing involved 10 s sprints at varied cadences with 4 min rest intervals. Pedaling cadences were set at low (60 rpm), moderate (80 and 100 rpm), and high (120 and 140 rpm) cadences. A regression model approach identified cIPF related to aerobic performance. **Results**: IPF at 80 and 120 rpm explained 49% of the variability in power output at VT1, 55% of the variability in power output at VT2, 65% of the variability in power output at maximal aerobic power (MAP), and 39% of the variability in VO_2_ max. The cycling economy was not explained by cIPF. **Conclusions**: This study highlights the significance of cIPF, particularly at moderate to high cadences, as a determinant of aerobic-related variables in trained triathletes. Cycling cIPF should be tested to understand an athlete’s profile during crank cycling, informing better practice for training specificity and ultimately supporting athletes in achieving optimal performance outcomes in competitive cycling events.

## 1. Introduction

Sports performance research focuses on providing coaches and athletes with information to inform better practice [1]. Various features govern the performance of individual cyclists in elite competitions, such as the cyclists’ physiological and morphological features, as well as cognitive skills [1]. Cycling performance, such as in triathlons, is influenced by physiological and mechanical factors such as maximum oxygen consumption (VO_2_ max), maximum aerobic power (MAP, i.e., the minimum power output that elicits VO_2_ max), cycling economy, physiological thresholds (e.g., power output at ventilatory thresholds, pVT), and neuromuscular aspects (e.g., strength/force) [2]. Interestingly, differences in these physiological characteristics could explain 40% of the variance between international cyclists’ finishing times [1]. Regarding neuromuscular aspects, the ability to sustain higher intensities for longer periods is directly related to an athlete’s MAP and physiological thresholds, which can be enhanced by adding strength training to the process [3,4]. Also, incorporating heavy strength training has an additive effect on time trial performance in well-trained cyclists [3]. Thus, strength capacity and training can enhance cycling aerobic performance.

Maximal leg strength significantly influences cycling performance in triathletes [5,6]. Cyclist athletes exhibit a strong correlation between maximal torque and lean leg volume, suggesting that greater leg strength contributes to enhanced cycling power and performance [7]. Furthermore, maximal leg-strength training improved cycling economy in previously untrained individuals, indicating that neuromuscular adaptations from strength training can enhance muscle force production and cycling performance [8,9]. Nevertheless, the effects of resistance training on muscle force are specific to the contraction velocities used in training [10]. Thus, an increase in the proportion of type IIa fibers at the expense of type IIX fibers has been observed in elite cyclists, contributing to improved time trial performance [3]. Accordingly, assessments of muscle strength help prescribe and monitor training loads in cyclists. Some methods include repetition maximum (RM), joint isokinetic tests, and indirect estimates [10,11,12]. However, their specificity for cycling’s dynamic force application and competitive cadences is lacking.

Several studies indicate that isokinetic strength, particularly in the lower limbs, positively correlates with cycling performance metrics such as power output and endurance [10,13]. For instance, one study characterizes lower body muscle strength among high-level cyclists and examines the relationship between isokinetic muscle strength and cycling sprinting power [13]. Their result suggests that enhanced isokinetic strength can improve cycling power output during competitive scenarios. Also, isokinetic muscular strength is relevant for triathletes and cyclists aiming to progress to higher competitive classes, indicating that isokinetic strength is a critical factor in overall cycling performance [14,15]. Specific isokinetic equipment uses electromagnetic brakes to determine pedaling torque and power output. Different muscle groups work systematically and coordinate to generate and direct power from the human body to the crank while cycling at different cadences. Thus, mono-articular muscles generate positive work, whereas the biarticular muscles regulate force transmission [16]. Nevertheless, the influence of cycling isokinetic peak force (cIPF) at different cadences on maximum and submaximal aerobic performance-related variables in cycling is not described. Determining and evaluating muscle strength under conditions of physical complexity specific to cycling could open new perspectives for assessing, monitoring, and prescribing strength training.

This study aims to determine the influence of the cIPF at different cadences on aerobic performance-related variables in trained triathletes.

## 2. Materials and Methods

### 2.1. Participants

A total of 14 triathletes were recruited, of which only 11 met the inclusion criteria and were classified as trained according to McKay et al. [17]. The inclusion criteria were maintaining a triathlon training time of ≥5 years, being without musculoskeletal injuries during the last 6 months, being ≥18 years old, and having a training frequency of at least 3 times per week. A sample of 10 subjects allows for the detection of a correlation coefficient of r = 0.7, with a statistical power of 80% and an alpha value of 5%. A loss rate of 10% was added to the initial sample calculation, leaving a final sample of n = 11. The sample was calculated through the G* Power statistical program (version 3.1.9.7). Before the evaluations, the subjects signed an informed consent document approved by the Scientific Ethics Committee of Finis Terrae University (ID: 22-053). This research was carried out within the framework of the Declaration of Helsinki agreed upon by the “World Medical Association”. The description of the participants is presented in Table 1.

### 2.2. Data Collection and Procedures

Subjects visited the laboratory on 3 occasions. All physical evaluations were randomized. In visit 1, the signing of the informed consent, the body composition assessment, and one of the physical tests were carried out. For visits 2 and 3, only one physical test was carried out in each of them. Each test was performed at least 48 h apart. All subjects were evaluated in a period not exceeding ~2 weeks to avoid time being a factor that interferes with the evaluated variables.

### 2.3. Estimation of the Body Composition

The anthropometric profile was realized according to the norms of the advancement of kinanthropometry as previously described [18]. The variables evaluated were weight, height, 8 skin folds (triceps, subscapular, biceps, suprailiac, abdominal, thigh, and calf), 6 bone diameters (acromion, iliac crest, transverse, anteroposterior chest, humeral, and femoral), and 9 circumferences (arm in relaxation and flexed in tension, maximum forearm, thorax, waist, maximum and medial thigh, and maximum calf). The body mass was evaluated with an electronic balance (SECA, accuracy 0.01 kg), the weight with an electronic stadiometer (SECA, accuracy 0.01 m), and the anthropometrics variables with the Health & Performance^®^ kit (Health & Performance^®^, Valparaíso, Chile).

### 2.4. Maximal Oxygen Uptake (VO_2_ max)

Subjects were tested on their bicycle mounted on an electromagnetic ergometer (Cyclus2, Leipzig, Germany). The cycle ergometer is programmed by entering data from the bicycle, the longitude of the crank, the smaller numbers of the pinion gear, and the biggest number of pinions of the plate. In addition, the athlete’s data (weight and height) were collected. This was applied for each of the physical evaluations. Before all the evaluations, the triathletes performed a warm-up of pedaling at 100 watts with a cadence of 90 ± 5 rpm for 10 min. The maximal oxygen consumption test began with an initial load of 100 watts and a cadence of 90 ± 5 rpm with increments of 25 watts every 1 min until exhaustion. Gas exchange was recorded continuously with a stationary breath-to-breath gas analyzer (Cortex Metalyzer 3B, Leipzig, Germany) previously calibrated according to the manufacturer’s recommendations before each test. The VO_2_ max was determined with three criteria: absolute VO_2_ max in the last two stages with modifications < 150 mL/min; RER ≥ 1.16; or voluntary withdrawal from the test. Also, ventilatory thresholds 1 (VT1) and 2 (VT2) were identified according to the following criteria [19]:

- VT1 (i.e., first physiological threshold): the intensity that causes the first systematic rise in the ventilatory equivalent of oxygen (VE/VO_2_) without a concurrent rise in the ventilatory equivalent of carbon dioxide (VE/VCO_2_).
- VT2 (i.e., second physiological threshold): the intensity that causes a concomitant rise in VE/VO_2_ and VE/VCO_2_ and a fall in end-tidal CO_2_ (PETCO_2_).

The cycling economy was calculated as previously described [19]. Briefly, the energy cost of pedaling (ECP) was determined as the total VO_2_ equivalent (mLO_2_/min) divided by the power output generated (W) at ventilatory thresholds intensities and maximal aerobic power (MAP):


(1) $$\mathrm{ECP}({\mathrm{mLO}}_{2}/W)=\frac{\mathrm{oxygen}\;\mathrm{consumption}}{\mathrm{power}\;\mathrm{output}}$$

### 2.5. Evaluation of the Cycling Isokinetic Peak Force

**Figure 1:**
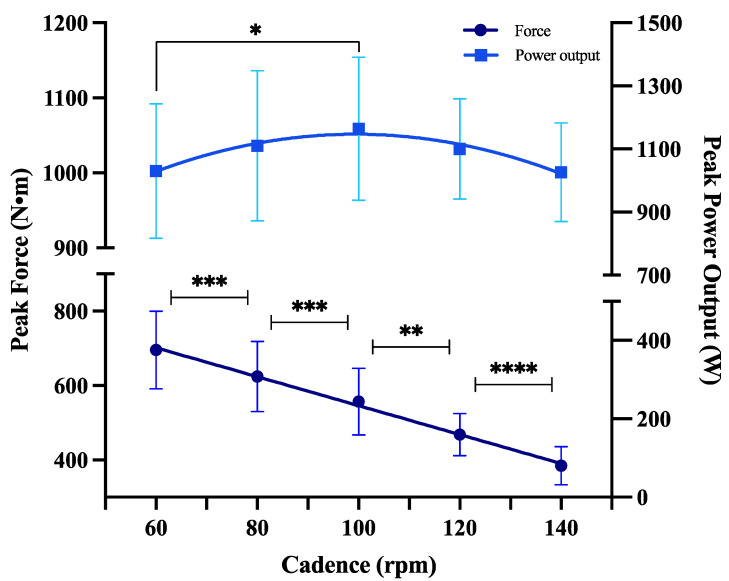
Force–velocity–power values according to pedaling cadence. * *p* ≤ 0.05; ** *p* ≤ 0.01; *** *p* ≤ 0.001; **** *p* ≤ 0.0001.

The evaluation of the isokinetic peak force (cIPF) in a cycle ergometer (Cyclus 2, Leipzig, Germany) consisted of sprints of 10 s at maximum intensity with 4 min of active pause at <60 rpm without load between each attempt (Figure 1). To cover the entire force spectrum as a function of pedaling cadence, five cadences were established and classified as follows [20]:

- Low cadence: 60 rpm (cIPF_60_).
- Moderate cadence: 80 (cIPF_80_) and 100 rpm (cIPF_100_).
- High cadence: 120 (cIPF_120_) and 140 rpm (cIPF_140_).

All tests were always performed in a sitting position and with hands on the handlebars. With these data, the force–velocity profile (FVP) in pedaling was calculated, representing the force–velocity and power–velocity relationships that the neuromuscular system of the lower extremities is capable of generating [21] (Figure 1).

### 2.6. Statistical Analysis

All data were expressed as mean ± standard deviation (SD) or 95% confidence intervals (CIs). Data normality was initially confirmed by Shapiro–Wilk tests. Stepwise linear regression analysis was performed to determine the effect of cIPF at different cadences on cycling aerobic-related variables. Before these analyses, a collinearity diagnostic procedure was implemented to reduce possible multicollinearity problems among predictor variables (VIF ≤ 5). A *p*-value threshold of 0.1 was used, meaning variables were included or removed from the model if their *p*-value was below or above this threshold. The linear regressions were performed with MAP, pVT2, pVT1, and VO_2_ max as the dependent variables and cIPF at different cadences as the independent variables. Adjusted coefficient of determination (R^2^) and root-mean-square error (RMSE) comprise the regression results. The effect size (ES) for multiple linear regressions was calculated using Cohen’s f^2^ [22]. The following threshold values for ES reported as f^2^ were employed: ≥0.02 as small, ≥0.15 as medium, and ≥0.35 as large. The ES for correlation was calculated using Cohen’s r. The following threshold values for ES reported as r were employed: ≥0.1 as small, ≥0.3 as medium, ≥0.5 as large, ≥0.7 as very large, and, ≥0.9 as extremely large [23]. Stata 14 (Release 18. College Station, TX, USA: StataCorp LLC) software was used for these analyses.

## 3. Results

The main results of the physiological variables of our study are shown in Table 2 and Figure 1. The applied linear regressions showed that cIPF at moderate (cIPF_80_) and high cadence (cIPF_120_) are the main determinants of aerobic performance-related variables (Table 3, Table 4, Table 5 and Table 6). As only 11 participants met the inclusion criteria, a subsequent post hoc power analysis [24] indicated that the current study achieved an overall statistical power of 78.5%.

### 3.1. Cycling Isokinetic Force Variables Related to the Power Output at VT1

When examining the cIPF variables influencing power output at VT1, cIPF_80_ and cIPF_120_ were identified as the main factors (Table 3). The stepwise linear regression model explained 49% of the variability in power output at VT1 (large effect).

### 3.2. Cycling Isokinetic Force Variables Determining Power Output at VT2

In analyzing cIPF variables affecting power output at VT2, cIPF_80_ and cIPF_120_ emerged as the primary determinants (Table 4). The stepwise linear regression model accounted for 55% of the variability in power output at VT2, indicating a large effect.

### 3.3. Cycling Isokinetic Force Variables Determining MAP

When investigating which cIPF variables influenced MAP, cIPF_80_ and cIPF_120_ were highlighted as the key contributors (Table 5). The stepwise linear regression model explained 65% of the variance in power at MAP, representing a large effect size.

### 3.4. Cycling Isokinetic Force Variables Determining VO_2_ max

In evaluating the cIPF variables impacting VO_2_ max, only cIPF_80_ was identified as the main factor (Table 6). The stepwise linear regression model explained 39% of the variability in VO_2_ max (large effect).

### 3.5. Relationship Between ∆cIPF_80_—cIPF_120_ and Aerobic Performance-Related Variables

**Figure 2:**
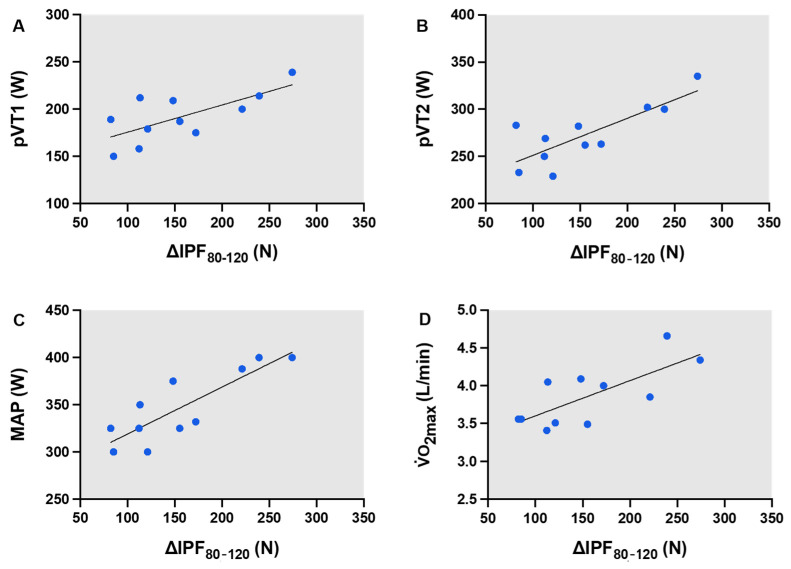
Association between ∆cIPF_80_—cIPF_120_ and aerobic related-performance variables (pVT1 (**A**), pVT2 (**B**), MAP (**C**), and VO_2_ max (**D**)).

The regression models consistently show that cIPF_80_ is positively associated and cIPF_120_ is negatively associated with the aerobic variables (positive and negative coefficients, respectively). The delta between both forces (i.e., ∆cIPF_80_—cIPF_120_; Figure 2) showed a stronger correlation than when examining cIPF_80_ and cIPF_120_ individually (Table 7).

A correlation analysis was performed to understand the delta between both forces and performance-related variables. The results show a very large association between ∆cIPF_80_—cIPF_120_ and pVT1 (r = 0.70; 95% CI (0.17 to 0.91); *p* = 0.02 *; Figure 2A), between ∆cIPF_80_—cIPF_120_ and pVT2 (r = 0.79; 95% CI (0.37 to 0.94); *p* = 0.00 **; Figure 2B), between ∆cIPF_80_—cIPF_120_ and MAP (r = 0.84; 95% CI (0.49 to 0.95); *p* = 0.00 **; Figure 2C), and between ∆cIPF_80_—cIPF_120_ and VO_2_ max (r = 0.74; 95% CI (0.25 to 0.92); *p* = 0.00 **; Figure 2D). Therefore, a higher delta indicates higher aerobic performance. There was no association between ∆cIPF_80_—cIPF_120_ and ECP variables.

## 4. Discussion

The main findings of this study highlight the implication of cIPF, particularly at moderate and high cadences, as a determinant of aerobic-related performance variables in trained triathletes. Determining and evaluating muscle strength under these conditions opens new perspectives for assessing, monitoring, and prescribing strength training.

### 4.1. Cycling Isokinetic Force and Submaximal Aerobic Cycling Performance

#### 4.1.1. Performance at VT1 and VT2

Our results show that cIPF_80_ and cIPF_120_ have a large effect, explaining 49% of the power output variability at VT1 and 55% at VT2. An interesting observation is that cIPF_80_ shows a positive association, while cIPF_120_ exhibits a negative association with aerobic variables (indicated by positive and negative coefficients, respectively). Remarkably, our results show that the delta between both forces best correlates with aerobic variables, not the cIPF separately. In general, cIPF_80_ is not positively correlated, and cIPF_120_ is not negatively associated with the aerobic performance-related variables (Table 7). This means that the relationship between both explains aerobic performance. Thus, ∆cIPF_80_—cIPF_120_ could be related to the profile of each athlete [25], where athletes with a greater difference between these forces tend to have better aerobic variables. A potential explanation is that diverse muscle groups perform systematically and coordinate to develop and produce power from the human body to the crank during cycling. Thus, mono-articular muscles are mainly involved in generating positive work, whereas the biarticular muscles are responsible for regulating force transmission. Factors such as cadence can alter muscle recruitment patterns’ characteristics [16]. Accordingly, the pedaling cadence may influence the fiber-type recruitment pattern. Fewer fast-twitch (type II) muscle fibers, compared with slow-twitch (type I) muscle fibers, are recruited when the pedal cadence is increased from 50 to 100 rpm [26]. The force demands of pedaling, rather than the velocity of contraction, determines the type of muscle fibers recruited [27]. However, this applies when attempting to maintain a given power output. In our case, the goal is to exert the greatest possible force at each cadence so that the recruitment pattern may differ. In this sense, greater forces at lower speeds could be related to higher recruitment of oxidative fibers (I and IIa). However, this must be corroborated in future studies since the effects of resistance training on muscle force are specific to the contraction velocities used in training [10]. Also, an increase in type IIa proportion at the expense of type IIX fibers has been observed in elite cyclists and can contribute to improved time trial performance [3]. Overall, to understand an athlete’s aerobic performance, cycling isokinetic tests at 80 and 120 should be performed to understand the athlete’s profile.

#### 4.1.2. Performance at ECP

Our results did not show a relationship between strength levels and the cycling economy. Several mechanical factors could influence the cycling economy, including biomechanical efficiency, muscle fiber composition, and pedal mechanics [28,29]. Also, this could be influenced by the athletes’ strength or training level [30]. Our results do not align with other studies that state that fiber-type recruitment and cycling efficiency appear to be linked with muscle contraction velocity. At 80 rpm, type I muscle fibers of the vastus lateralis contract closer to their peak efficiency contraction velocity than type II muscle fibers [27]. However, strength at any cadence was not related to the economy in our study. Another explanation is the methodology used to assess the economy. It has been suggested that the cycling economy needs to be measured by the same traditional method used in running (i.e., short, 3–5 min, submaximal bouts of exercise) [30]. However, we evaluated it during the incremental test used to assess VO_2_ max as previously described in rowing [19], which could interfere with assessing steady-state and representative pedaling costs. Finally, muscle recruitment control is less developed in triathletes than in trained cyclists, suggesting that multidiscipline training may interfere with neuromuscular adaptations such as the cycling economy in triathletes [31]. Further studies are needed to confirm whether IPF is unrelated to the cycling economy.

### 4.2. Cycling Isokinetic Force and Aerobic Cycling Performance at MAP and VO_2_ max

The stepwise linear regression model explained 64% of the power output variability at MAP (large effect) and 39% of the variability in VO_2_ max (large effect). Similarly, a study showed that knee strength at 60º/s and the percentage of type I fibers could explain up to 40% of the variation in VO_2_ peak and MAP. Notably, the percentage of type I fibers contributed only about 10% to VO_2_ peak and MAP [4]. These findings align with our results concerning forces applied at low/moderate velocities. In our study, cycling isokinetic pedaling peak at 80 rpm could account for including type IIa fibers, which likely impact maximal aerobic performance, given that isokinetic forces at 60º/s are linked to type IIa fibers [4]. Also, our results are concordant with a previous study in terms of the fact that it seems that low-cadence interval training (60–70 rpm) is more effective than high-cadence (110–120 rpm) training in improving the aerobic performance of well-trained competitive cyclists [20]. The negative association between cIPF_120_ and aerobic performance may be due to the type of force, as adaptations depend on the velocity used [10] where factors such as cadence can alter muscle recruitment patterns’ characteristics [16]. Thus, concurrent endurance and heavy strength training can increase MAP or time to exhaustion at MAP [32,33,34]. However, this positive effect on cyclists was not observed when using explosive strength training [30]. The results suggest that strength at moderate isokinetic pedaling velocities should be stimulated when aiming to improve maximal aerobic variables.

### 4.3. Limitations

One limitation is the methodology used to assess the economy. It has been suggested that the cycling economy needs to be measured during submaximal bouts of exercise (e.g., 3–5 min) [30]. Another limitation is the sample size. Additional studies with a larger sample should be conducted to confirm our findings.

## 5. Conclusions

Due to this isokinetic cycling test’s specificity, our results open new perspectives in sports performance research, focusing on providing valuable information to inform better practice. The results of our preliminary study show promising tools for determining, monitoring, and prescribing muscle strength under conditions of physical complexity specific to cycling. The data provided can give us reliable information on the sport’s specific strength, aerobic capacity, and power development, but not on the cycling economy. Both pedaling isokinetic forces, cIPF_80_ and cIPF_120_, should be tested to understand an athlete’s profile comprehensively and specifically during crank cycling. Training at low/moderate cadences (e.g., cIPF_80_) could be related to better improvements in aerobic performance, potentially offering guidance for optimizing training strategies and decision-making in long-term training programs. To expand the usefulness of this isokinetic pedaling test, and due to the specific characteristics of triathletes, further research is required with higher-level triathletes, cyclists from different disciplines, and larger sample sizes.

### Practical Implications

Coaches and sports scientists will be able to collect vital information to determine and program muscular and aerobic training, considering specific data on complex physical qualities.

## Figures and Tables

**Table 1:** General characteristics of the athletes.

Variable	Mean ± SD
Age (years)	33 ± 9.8
Weight (kg)	73.9 ± 6.8
Height (cm)	173.1 ± 5.0
Sum of folds (∑ 8)	70.3 ± 25.2
Muscle mass (kg)	38.5 ± 4.2
Muscle mass (%)	51.7 ± 2.8
Fat mass (kg)	15.7 ± 3.6
Fat mass (%)	20.6 ± 3.9

**Table 2:** Descriptive aerobic and cycling isokinetic values.

Variable	Mean ± SD
VO_2_ max (L/min)	3.87 ± 0.40
MAP (W)	347 ± 38
ECP max (mLO_2_ × W)	11.2 ± 0.7
VO_2_ at pVT2 (L/min)	3.33 ± 0.36
pVT2 (W)	273 ± 31
ECP at pVT2 (mLO_2_ × W)	12.2 ± 0.8
VO_2_ at pVT1 (L/min)	2.56 ± 0.35
pVT1 (W)	192 ± 26
ECP at pVT1 (mLO_2_ × W)	13.4 ± 1.0
cIPF_60_ (N × m)	695 ± 104
cIPF_80_ (N × m)	625 ± 95
cIPF_100_ (N × m)	557 ± 90
cIPF_120_ (N × m)	468 ± 57
cIPF_140_ (N × m)	385 ± 52
Slope FVP	−3.91 ± 1.22

MAP: maximal aerobic power; ECP: energy cost of pedaling; pVT: power output at the ventilatory threshold; cIPF: cycling isokinetic peak force; FVP: force–velocity profile.

**Table 3:** Cycling isokinetic force variables determining power output at VT1.

Variable	Coefficient (B)	Std. Err.	t	*p*	95% CI		Adj. R-Squared	Root MSE	Prob > F	Cohen f^2^	Effect Size
					Lower Limit	Upper Limit					
cIPF_120_	−0.47	0.15	−2.98	0.017	−0.83	−0.10	0.49	18.57	0.03	1.0	Large
cIPF_80_	0.32	0.09	3.38	0.010	0.10	0.53					
Intercept	213.19	49.04	4.35	0.002	100.09	326.28					

cIPF: cycling isokinetic peak force; CI: confidence interval; MSE: mean square error. *p*-value for excluded variables: cIPF_60_: *p* = 0.8936; cIPF_100_: *p* = 0.6500; cIPF_140_: *p* = 0.3083.

**Table 4:** Cycling isokinetic force variables determining power output at VT2.

Variables	Coefficient (B)	Std. Err.	t	*p*	95% Conf. Interval		Adj. R-Squared	Root MSE	Prob > F	Cohen f^2^	Effect Size
					Lower Limit	Upper Limit					
cIPF_120_	−0.45	0.17	−2.56	0.034	−0.86	−0.04	0.55	20.96	0.02	1.3	Large
cIPF_80_	0.40	0.10	3.78	0.005	0.15	0.64					
Intercept	235.03	55.34	4.25	0.003	107.41	362.64					

cIPF: cycling isokinetic peak force; CI: confidence interval; MSE: mean square error. *p*-value for excluded variables: cIPF_60_: *p* = 0.9703; cIPF_100_: *p* = 0.5755; cIPF_140_: *p* = 0.7364.

**Table 5:** Cycling isokinetic force variables determining maximal aerobic power.

Variable	Coefficient (B)	Std. Err.	t	*p*	95% Conf. Interval		Adj. R-Squared	Root MSE	Prob > F	Cohen f^2^	Effect Size
					Lower Limit	Upper Limit					
cIPF_120_	−0.56	0.18	−3.01	0.017	−1.00	−0.13	0.65	22.28	0.01	1.9	Large
cIPF_80_	0.51	0.11	4.50	0.002	0.24	0.77					
Intercept	295.70	58.83	5.03	0.001	160.02	431.37					

cIPF: cycling isokinetic peak force; CI: confidence interval; MSE: mean square error. *p*-value for excluded variables: cIPF_60_: *p* = 0.9119; cIPF_100_: *p* = 0.6128; cIPF_140_: *p* = 0.8609.

**Table 6:** Cycling isokinetic strength variables determining maximal oxygen consumption.

Variable	Coefficient (B)	Std. Err.	t	*p*	95% Conf. Interval		Adj. R-Squared	Root MSE	Prob > F	Cohen f^2^	Effect Size
					Lower Limit	Upper Limit					
cIPF_80_	0.003	0.001	2.77	0.022	0.001	0.005	0.39	0.31	0.02	0.7	Large
Intercept	2.064	0.65	3.14	0.012	0.575	3.552					

cIPF: cycling isokinetic peak force; CI: confidence interval; MSE: mean square error. *p*-value for excluded variables: cIPF_60_: *p* = 0.8623; cIPF_100_: *p* = 0.1342; cIPF_120_: *p* = 0.3058; cIPF_140_: *p* = 0.8808.

**Table 7:** Association between cIPF_80_ and cIPF_120_ with the aerobic performance-related variables.

Variables	r	95% CI	*p*
VT1—cIPF_80_	0.38	−0.27 to 0.80	0.240
VT2—cIPF_80_	0.59	−0.01 to 0.88	0.053
MAP—cIPF_80_	0.63	0.05 to 0.89	0.035 *
VO_2_ max—cIPF_80_	0.67	0.13 to 0.90	0.021 *
VT1—cIPF_120_	−0.14	−0.68 to 0.49	0.661
VT2—cIPF_120_	0.09	−0.53 to 0.65	0.784
MAP—cIPF_120_	0.10	−0.52 to 0.66	0.751
VO_2_ max—cIPF_120_	0.29	−0.36 to 0.76	0.375

CI: confidence interval; MAP: maximal aerobic power; pVT: power output at the ventilatory threshold; cIPF: cycling isokinetic peak force. * *p* ≤ 0.05.

## Data Availability

Data supporting this article are available from the corresponding author upon reasonable request.
